# *Potamophylaxhumoinsapiens* sp. n. (Trichoptera, Limnephilidae), a new species from the Sharr Mountains, Republic of Kosovo

**DOI:** 10.3897/BDJ.11.e97969

**Published:** 2023-01-11

**Authors:** Halil Ibrahimi, Astrit Bilalli, Agim Gashi, Linda Grapci Kotori, Valentina Slavevska Stamenkovič, Donard Geci

**Affiliations:** 1 Department of Biology, Faculty of Mathematics and Natural Sciences, University of Prishtina “Hasan Prishtina”, Prishtina, Kosovo Department of Biology, Faculty of Mathematics and Natural Sciences, University of Prishtina “Hasan Prishtina” Prishtina Kosovo; 2 Faculty of Agribusiness, University of Peja “Haxhi Zeka”, Peja, Kosovo Faculty of Agribusiness, University of Peja “Haxhi Zeka” Peja Kosovo; 3 Institute of Biology, Faculty of Natural Sciences and Mathematics, Skopje, Macedonia Institute of Biology, Faculty of Natural Sciences and Mathematics Skopje Macedonia

**Keywords:** endemic species, Western Balkans, *Potamophylaxwinneguthi* Species Group, microscale distribution

## Abstract

**Background:**

The Sharr Mountains are one of the most important hotspots of terrestrial and freshwater biodiversity in the Balkan Peninsula, with many endemic and rare species. The caddisfly studies in this area increased during the past years, although insufficiently investigated areas still remain.

**New information:**

In this paper, we describe a new species, *Potamophylaxhumoinsapiens* sp. n. from the Sharr Mountains in the Republic of Kosovo, which is morphologically closest to *Potamophylaxidliri* Ibrahimi, Bilalli & Kučinić, 2022 and *Potamophylaxjuliani* Kumanski, 1999. The males of the new species differ from all known species of the *Potamophylaxwinneguthi* Species Group by their uniquely-shaped parameres, which are long, bulbous in their basal half and thin in the remaining length, with a bunch of very thin and long, hair-like spines, grouped uniformly at the apex. The new species further differs from its most similar congeners by its very wide distance between the dorsal and ventral edges of the apical part of inferior appendages in lateral view. The new species was found at three localities from 1416 to 1505 m a.s.l.

Similar to the other species of the *Potamophylaxwinneguthi* Species Group, which have very narrow distribution areas, we posit that *Potamophylaxhumoinsapiens* sp. n. is a microendemic of the Sharr Mountains. The new species is the second known caddisfly species occurring only in the Kosovan part of the Sharr Mountains.

## Introduction

The knowledge about the caddisflies of Kosovo has increased significantly over the past decade. Only few species were known before 2011 (e.g. Pongrácz 1923, Marinković-Gospodnetić 1975, Marinković-Gospodnetić 1980, Malicky 1986) and the number has increased to 169 now (e.g. Ibrahimi et al. 2012, Ibrahimi et al. 2014, Ibrahimi et al. 2015, Ibrahimi et al. 2016, Karaouzas et al. 2018, Ibrahimi et al. 2019a, Ibrahimi et al. 2019b, Ibrahimi et al. 2022). Despite this, there are still areas which are not completely investigated and new records, or even species, can still be expected from isolated habitats of different mountain ranges, such as the the Sharr Mountains, for example.

The Sharr Mountains are one of the most important hotspots of freshwater and terrestrial biodiversity in the Balkan Peninsula, with many endemic and rare plant and animal species (e.g. Ibrahimi et al. 2016, Ibrahimi and Vehapi 2017, Komnenov 2017, Grapci-Kotori et al. 2022, Halilaj et al. 2021). This mountain range is shared between Kosovo and North Macedonia, while a smaller portion lies in Albania as well. The largest part of the Sharr Mountains lies in Kosovo, with a significant portion being a protected area since 1980. During 2014, the then existing National Park of the Sharr Mountains extended into the Dragash Municipality and the total protected area in this mountain range now counts for 53,471.48 ha (Ministry of Environment and Spatial Planning, Republic of Kosovo 2015). Recently, a National Park was proclaimed in the part of the Sharr Mountains belonging the Republic of North Macedonia as well (Ministry of Environment and Spatial Planning, Republic of North Macedonia 2021).

In this paper, we describe a new species of the *Potamophylaxwinneguthi* Species Group from the Sharr Mountains in Kosovo and also discuss morphological, molecular and ecological features of the *Potamophylaxwinneguthi* Species Cluster.

## Materials and methods

**Fieldwork, identification and taxonomic work.** We collected adults of the new species with ultraviolet light traps. Nocturnal light trapping followed Malicky (2004). Specimens were stored directly into 90% ethanol. The collected material is deposited at the Department of Biology, Faculty of Mathematics and Natural Sciences, University of Prishtina “Hasan Prishtina”, Prishtinë, Kosovo. For comparative assessments of morphological features of *Potamophylaxhumoinsapiens* sp. n., we used specimens of *Potamophylaxjuliani* Kumanski, 1999, *Potamophylaxwinneguthi* Malicky, 1999 (in Kumanski and Malicky 1999), *Potamophylaxidliri* Ibrahimi, Bilalli & Kučinić, 2022 (in Ibrahimi et al. 2022) and *Potamophylaxcoronavirus* Ibrahimi, Bilalli & Vitecek, 2021 (in Ibrahimi et al. 2021), collected in Osogovo Mountain in Bulgaria, Zlatibor Mountain in Serbia, Jastrebac Mountains in Serbia and Bjeshkët e Nemuna in Kosovo, accordingly. For the lacking species, comparative assessment was done, based on literature (Kumanski and Malicky 1999, Malicky 2004).

Morphological characteristics of male terminalia of the new species were examined in specimens cleared in 10% potassium hydroxide (KOH). Nomenclature of male terminalia follows Nielsen (1957) for *Limnephilusflavicornis* (Fabricius, 1787) and Kumanski and Malicky 1999. Systematic nomenclature follows Morse (2023).

Morphological features of genitalia of *Potamophylaxhumoinsapiens* sp. n. were analysed from 12 male specimens by using the Olympus SZX16 stereomicroscope. Illustrations were prepared in Adobe Illustrator (version Creative Cloud 2018) by digitising pencil templates drawn in the pictures taken with Olympus SC50 camera.

**Figure 1.**
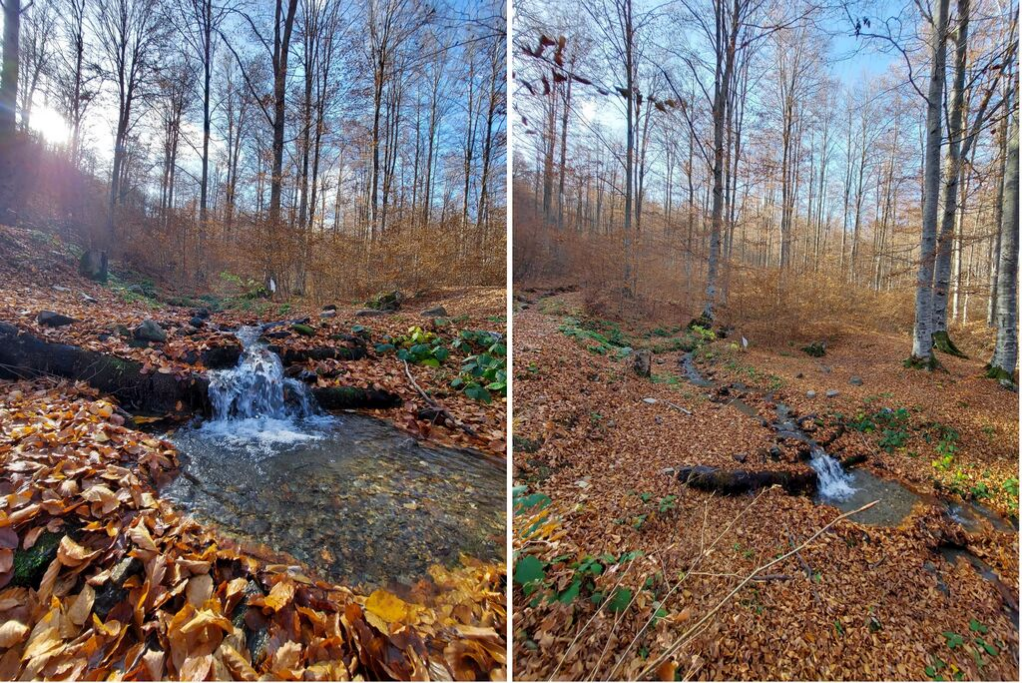
Type locality of *Potamophylaxhumoinsapiens* sp. n.: a tributary of the Lepenc River along the Brezovicë - Prevallë road.

Specimens of the new species were collected at three localities in the Sharr Mountains, belonging to the Shtërpce and Prizren Municipalities (Fig. 1)

## Taxon treatments

### Potamophylax humoinsapiens

Ibrahimi & Bilalli sp. n.

721160E4-BE2A-5F10-A8A4-8B4BA1A08408

95A49795-937A-419F-BA72-A85AD2C1C98E

#### Materials

- **Type status:** Holotype. **Occurrence:** recordedBy: Halil Ibrahimi, Astrit Bilalli; individualCount: 1; sex: male; lifeStage: adult; occurrenceID: 6E91A186-F8FF-5E67-A55C-F4BF514BD704; **Taxon:** class: Insecta; order: Trichoptera; family: Limnephilidae; genus: Potamophylax; nomenclaturalCode: ICZN; **Location:** continent: Europe; waterBody: Aegean Sea Basin; country: Kosovo; countryCode: XKS; municipality: Shtërpce; locality: main road towards Prevallë; verbatimLocality: sidestream of the Lepenc River; verbatimElevation: 1416; decimalLatitude: 42.172804; decimalLongitude: 20.969464; **Event:** samplingProtocol: UV light trap; year: 2021; month: 11; day: 12
- **Type status:** Paratype. **Occurrence:** recordedBy: Halil Ibrahimi, Astrit Bilalli; individualCount: 12; sex: males; lifeStage: adults; occurrenceID: 89E22BF4-8039-5F0B-A92B-6620CF80DFB8; **Taxon:** class: Insecta; order: Trichoptera; family: Limnephilidae; genus: Potamophylax; nomenclaturalCode: ICZN; **Location:** continent: Europe; waterBody: Aegean Sea Basin; country: Kosovo; countryCode: XKS; municipality: Shtërpce; locality: main road towards Prevallë; verbatimLocality: sidestream of the Lepenc River; verbatimElevation: 1416; decimalLatitude: 42.172804; decimalLongitude: 20.969464; **Event:** samplingProtocol: UV light trap; year: 2021; month: 11; day: 12
- **Type status:** Paratype. **Occurrence:** recordedBy: Halil Ibrahimi, Astrit Bilalli; individualCount: 20; sex: males; lifeStage: adults; occurrenceID: 97FDA7E5-5061-50AA-9C21-CE0ADE296551; **Taxon:** class: Insecta; order: Trichoptera; family: Limnephilidae; genus: Potamophylax; nomenclaturalCode: ICZN; **Location:** continent: Europe; waterBody: Aegean Sea Basin; country: Kosovo; countryCode: XKS; municipality: Shtërpce; locality: 2 km away from the main road Shtërpce - Prevallë; verbatimLocality: Lepenc River; verbatimElevation: 1505; decimalLatitude: 42.176299; decimalLongitude: 20.984170; **Event:** samplingProtocol: UV light trap; year: 2021; month: 11; day: 20
- **Type status:** Paratype. **Occurrence:** recordedBy: Halil Ibrahimi, Astrit Bilalli; individualCount: 7; sex: males; lifeStage: adults; occurrenceID: 68CC6836-CFAE-5D3A-8226-FA10865B105E; **Taxon:** class: Insecta; order: Trichoptera; family: Limnephilidae; genus: Potamophylax; nomenclaturalCode: ICZN; **Location:** continent: Europe; waterBody: Aegean Sea Basin; country: Kosovo; countryCode: XKS; municipality: Shtërpce; locality: few kilometers before Brezovica ski center; verbatimLocality: tributary of the Lepenc River; verbatimElevation: 1457; decimalLatitude: 42.185053; decimalLongitude: 21.006089; **Event:** samplingProtocol: UV light trap; year: 2021; month: 11; day: 12

#### Description

**Figure 2.**
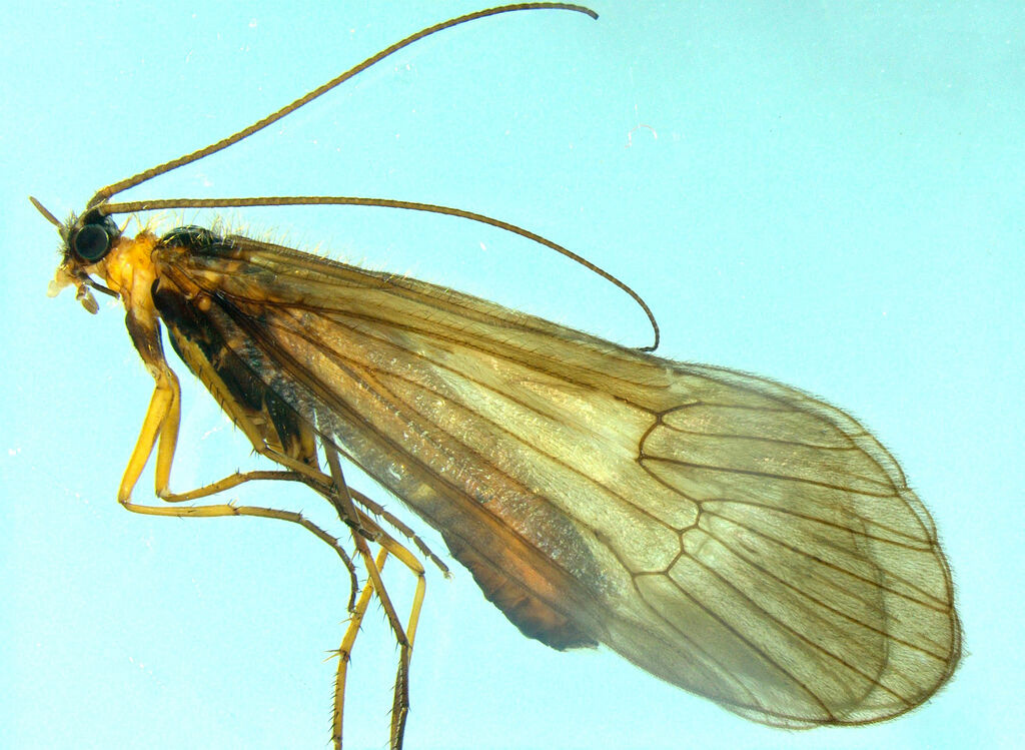
Male adult of *Potamophylaxhumoinsapiens* sp. n.

**Male.** General appearance (Fig. 2). Head and appendages brown, prothorax, sclerites of meso- and metathorax, coxae and femora dark brown to brown; tibiae and tarsi brown. Wings light brown with brown setae. Male maxillary palps each 3-segmented. Length of each forewing 14.9–15.9 mm. Spur formula 1-3-4. Antennae brown.

**Figure 3.**
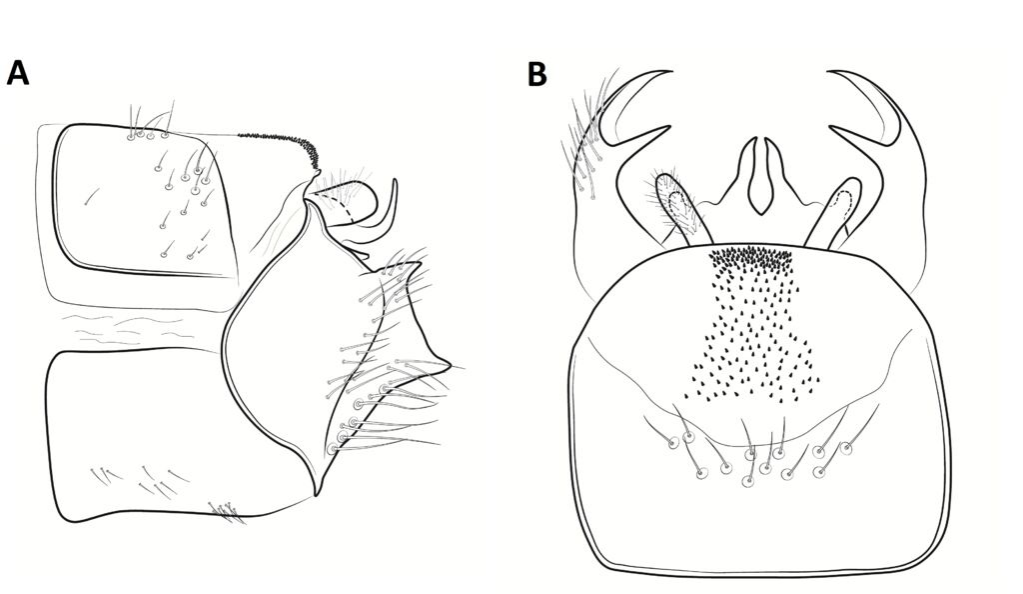
Male genitalia of *Potamophylaxhumoinsapiens* sp. n.: **A.** left lateral view; **B.** dorsal view.

**Figure 4.**
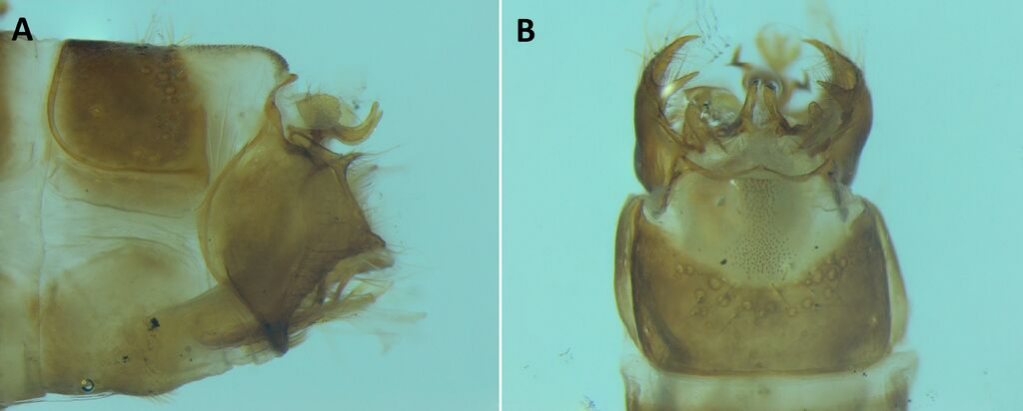
Male genitalia of *Potamophylaxhumoinsapiens* sp. n.: **A.** left lateral view; **B.** dorsal view.

**Figure 5.**
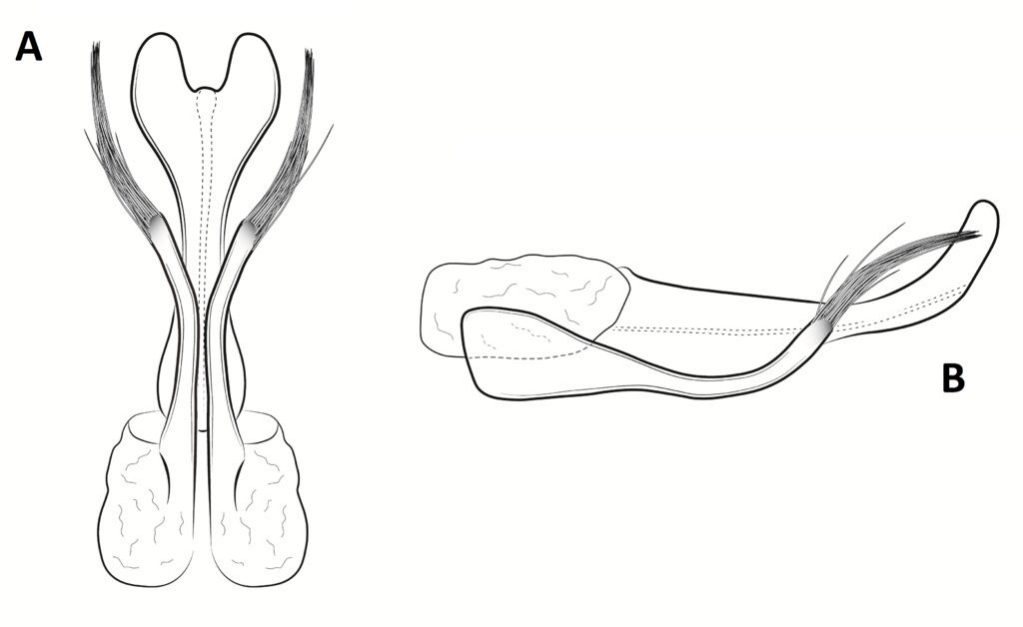
Aedeagus and parameres of the male genitalia of *Potamophylaxhumoinsapiens* sp. n.: **A.** ventral view; **B.** lateral view.

**Figure 6.**
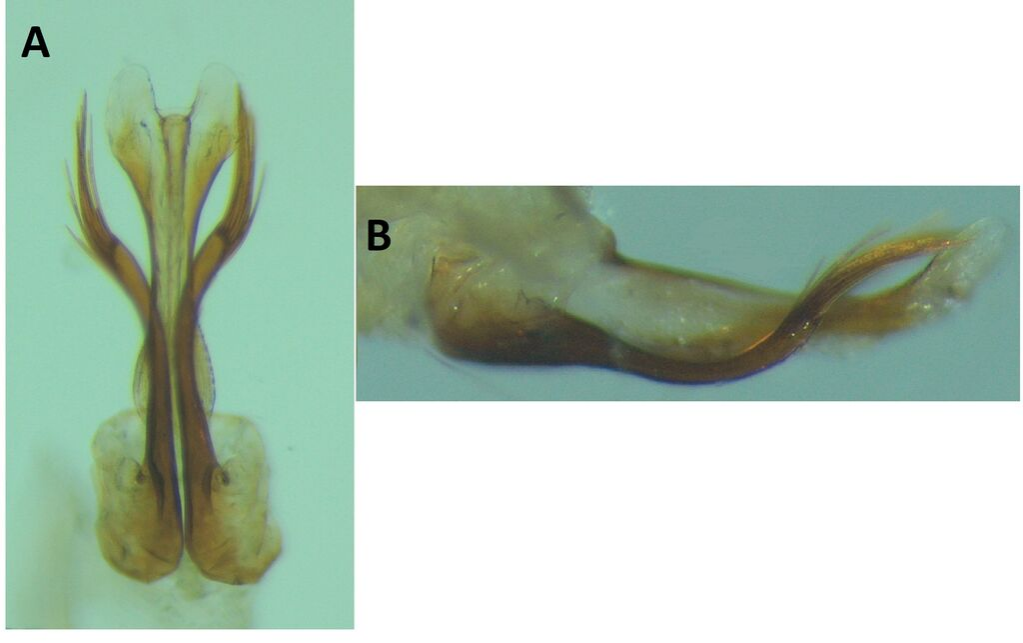
Aedeagus and parameres of the male genitalia of *Potamophylaxhumoinsapiens* sp. n.: **A.** ventral view; **B.** lateral view.

Male genitalia (Figs 3, 4, 5, 6). Tergite VIII generally light brown with only a few darker patches, in dorsal view roughly rectangular, with apical portion slightly narrower, several irregularly distributed setae concentrated on proximal sclerotised portion of segment VIII, spinate area located on semi-membranous distal portion of segment VIII with slightly wider proximal portion in dorsal view, elongated, covered by small black spines. Segment IX light brown with few darker patches, with narrow dorsal and ventral portions, laterally broad, convex anteriorly. Superior appendages light brown, in lateral view long, subrectangular, with rounded tips, covered with fine pale setae of medium length, base slightly narrower than the apex. Intermediate appendages, sickle-shaped with accuminate apex, strongly turned upwards. Inferior appendages with bifid apex, turned upwards, each with longer ventral edge. Phallic apparatus consisting of aedeagus of medium length and a pair of parameres. Aedeagus bulbous in ventral view, wide at tip, with bifid apex, apicomesal excision medium-U-shaped; parameres long, with very wide basal half and slender apical half in lateral view, bearing a bunch of apical thin and long hair-like spines, grouped uniformly.

#### Diagnosis

Males of the new species are most similar to those of *Potamophylaxidliri*. They have some resemblance with *P.coronavirus* and *P.juliani* as well and, thus, we compare it with all the three species (Figs 7, 8). The males of the new species differ from all known species of the *Potamophylaxwinneguthi* Species Group primarily by their uniquely-shaped parameres and inferior appendages. The *P.humoinsapiens* sp. n. male differs from that of its most similar congeners mainly in exhibiting the combination of the following characters: (1) in lateral view very long paramere shaft, bulbous in basal half, thin in the remaining length; (2) median ventral incision on paramere shaft in lateral view; (3) parallel set-up of the basal 2/3^rd^ of parameres on ventral view, with distal thirds diverging greatly from each other; (4) 30-40 very thin and long apical hair-like spines on parameres, grouped uniformly, curved and directed mesad in lateral view, almost reaching apex of the aedeagus in ventral view; (5) very wide distance between the dorsal and ventral corners of the apical part of inferior appendages, 2.1 times wider than in *P.coronavirus*, 1.4 times wider than in *P.idliri* and 1.2 times wider than in *P.juliani*; and (6) longer ventral edge of inferior appendages. The *P.idliri* male has long shaft of parameres, very narrow mesally, wide apically on lateral view; ventral incision on paramere shaft is located right after the basal third on lateral view; parallel set-up of the basal 1/3^rd^ of parameres, with the remaining length gradually diverging from each other on ventral view; 10-15 medium long and very thick apical spines of different sizes on parameres, grouped irregularly, directed mesad; longer ventral edge of inferior appendages on lateral view. The *P.juliani* male has a short shaft of parameres with wider basal half on lateral view without any incision; parallel set-up of parameres, only with apices diverging from each other on ventral view; medium long 5-10 medium thick apical spines on parameres; ventral and dorsal corners of inferior appendages parallel to each other, directed mesad on lateral view. The *P.coronavirus* male has a short shaft of parameres, slightly wider on basal half on lateral view; ventral margin of paramere shaft straight, without any incision on lateral view; parallel set-up of parameres, only with apices diverging from each other, on ventral view; short, thick 5-10 apical spines on parameres; ventral and dorsal corners of inferior appendages parallel to each other, directed mesad on lateral view.

#### Etymology

The species epithet is a combination of two Latin words, ‘humo’, which in English means ‘to cover with soil, to bury’ and ‘insapiens’ meaning ‘unwise’ and refers to the unwise and careless treatment of habitats of the new species, degraded greatly during the past years by hydropower plants and other activities. In some segments, the whole parts of the Lepenc River are ‘buried’ in large pipes.

#### Ecology

*Potamophylaxhumoinsapiens* sp. n. was found at three localities during 2021 in the Sharr Mountains, in the tributaries of the Lepenc River. All sampling sites are located inside a forested area. The substrates of streams close to the sampling sites were dominated by meso- to macrolithal substrate, surrounded by dense riparian vegetation. The species was captured only by ultraviolet light traps. The species was collected during October and November, implying it has an autumn flying period. All sampling sites are located in upstream sections of streams and rivers.

## Discussion

Two male specimens of *Potamophylaxhumoinsapiens* sp. n. have been found during 2009 in tributaries of the Lepenc and Lumbardhi i Prizrenit Rivers. The genetic distinction, calculated based on sequencing of the barcode region of the cytochrome *c* oxidase subunit I gene (*COI*) between *Potamophylaxhumoinsapiens* sp. n. and other species of the *Potamophylaxwinneguthi* Species Group, was found to be on par with those of the other morphologically recognised species in the group and especially the other five species of the *P.winneguthi* Species Cluster (Ibrahimi et al. 2021, Ibrahimi et al. 2022). These molecular analysis showed that the closest species to *Potamophylaxhumoinsapiens* sp. n. is *Potamophylaxidliri* from the Jastrebac Mountains in Serbia, with the p-distance of 4%. However, due to the low number of specimens, we refrained from describing it as a new species at that time. During 2021, we collected a large number of male specimens and, based on a large scale analysis of more than 40 male specimens, we realised that the differences in paramere shape, spine pattern, inferior appendages and other characters are stable and easily distinguishable from all other species of this Species Group.

Currently the *Potamophylaxwinneguthi* Species Cluster contains six species, all of them confined to certain mountains of the Balkan Peninsula. Species of this cluster have diversified in several characters of the male terminalia, the most visible being paramere shaft shape and size, paramere spine pattern and length and inferior appendages shape. Paramere shaft varies in length, width of basal and apical parts, as well as the curvature. Paramere spine pattern is especially diverse in this species complex. Spines vary from being very thick in *P.winneguthi*, medium thick in *P.juliani*, *P.idliri* and *P.coronavirus* and very thin and hair-like in *P.humoinsapiens* sp. n. Length of paramere spines varies from very short as 1/5^th^ of the total paramere shaft lengh in *P.juliani* and *P.coronavirus*, as 1/3^rd^ of paramere shaft in *P.idliri*, as half of paramere shaft in *P.humoinsapiens* sp. n. and as long as paramere shaft in *P.winneguthi*. In *P.haidukorum* Malicky, 1999 spines are lacking completely and paramere shaft is long and slender. The level of divergence in the female terminalia remains to be studied, as currently only females of *P.juliani*, *P.winneguthi* and *P.haidukorum* are described (Kumanski and Malicky 1999, Oláh and Kovács 2012, Oláh and Kovács 2013).

Species of the *Potamophylaxwinneguthi* Species Group are all found at isolated habitats at different mountain ranges of the Balkan Peninsula, usually at upstream segments of streams and rivers. These habitats have deteriorated heavily during the past decades by illegal logging, pollution and water extraction. Considering the fact that all species of this cluster up to now are known to be microscale endemics of certain mountain ranges, we posit that other new species will be found in the Balkans in future.

*Potamophylaxhumoinsapiens* sp. n. is the second known endemic caddisfly species occurring only in the Kosovan part of the Sharr Mountains, the first one being *Drusussharrensis* Ibrahimi, Kučinić & Vitecek, 2015. This part of the Sharr Mountains represents the spring and upstream area of two rivers, namely Lepenc and Lumbardhi i Prizrenit. During the past decade, both rivers have deteriorated heavily, primarily by construction of dams and hydropower plants, illegal logging and pollution. Some of these activities occur in the very vicinity of the freshwater ecosystems where the new species is found. During the sampling of 2021, the new species was not found at one of the localities where it was sampled during 2009. Although large areas of upstream segments of both rivers are within the borders of the protected area, more law enforcement activities in the field are needed in order to protect unique freshwater diversity of this part of the Sharr Mountains, including the newly-described species *Potamophylaxhumoinsapiens* sp. n.

## Supplementary Material

XML Treatment for Potamophylax humoinsapiens

## Figures and Tables

**Figure 7a.**
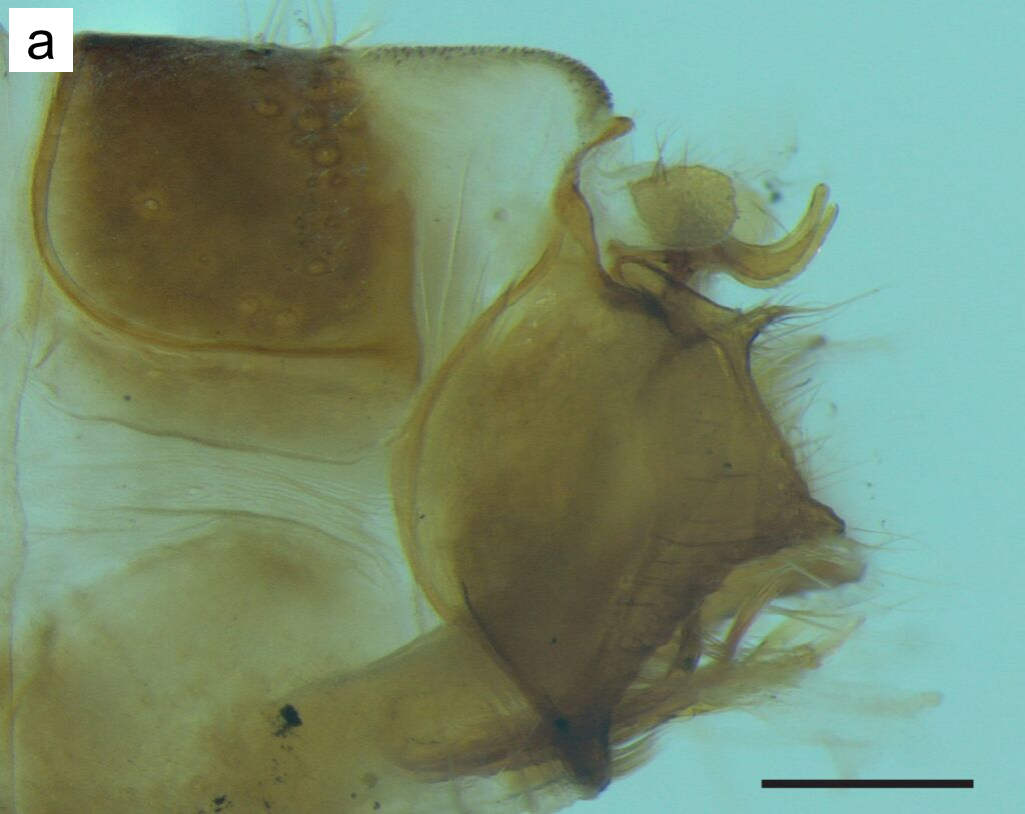
*Potamophylaxhumoinsapiens* sp. n.

**Figure 7b.**
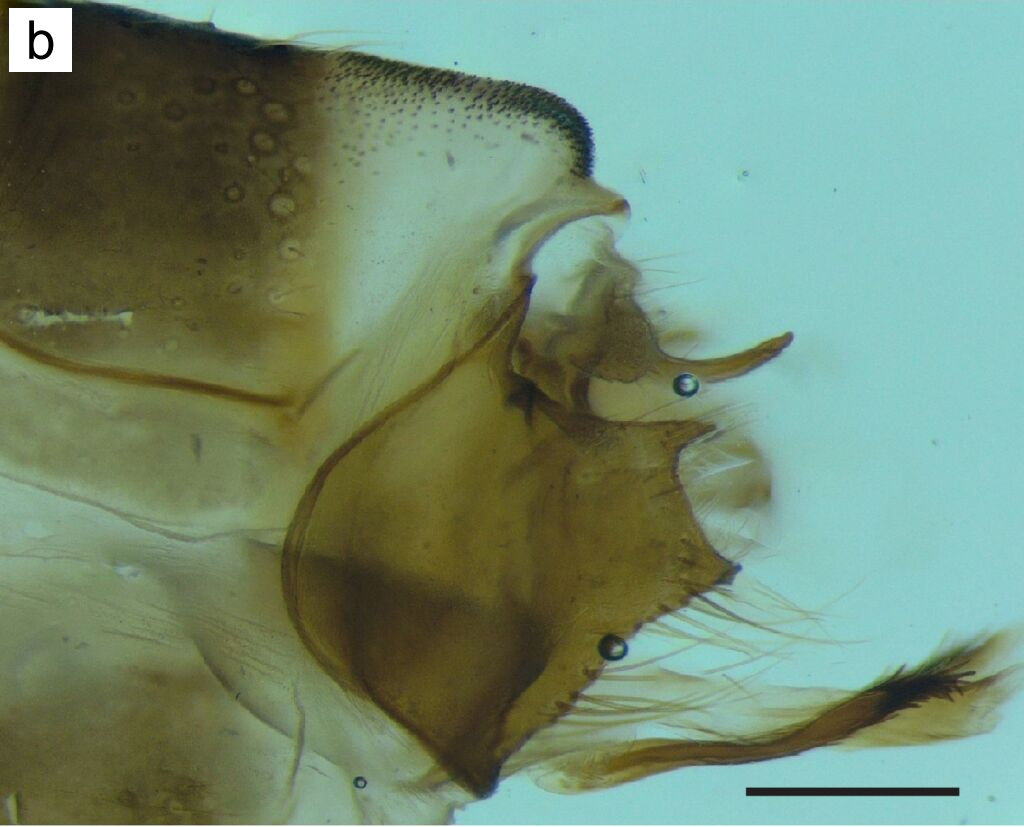
*Potamophylaxidliri* Ibrahimi, Bilalli & Kučinić, 2022.

**Figure 7c.**
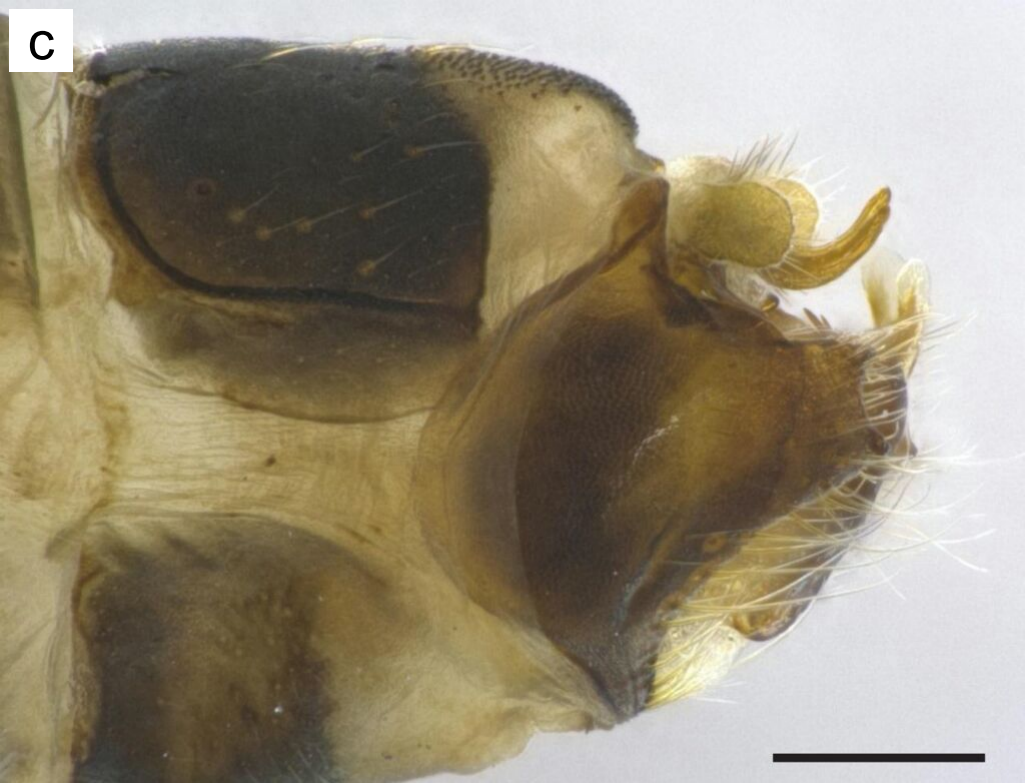
*Potamophylaxcoronavirus* Ibrahimi, Bilalli & Vitecek, 2021.

**Figure 7d.**
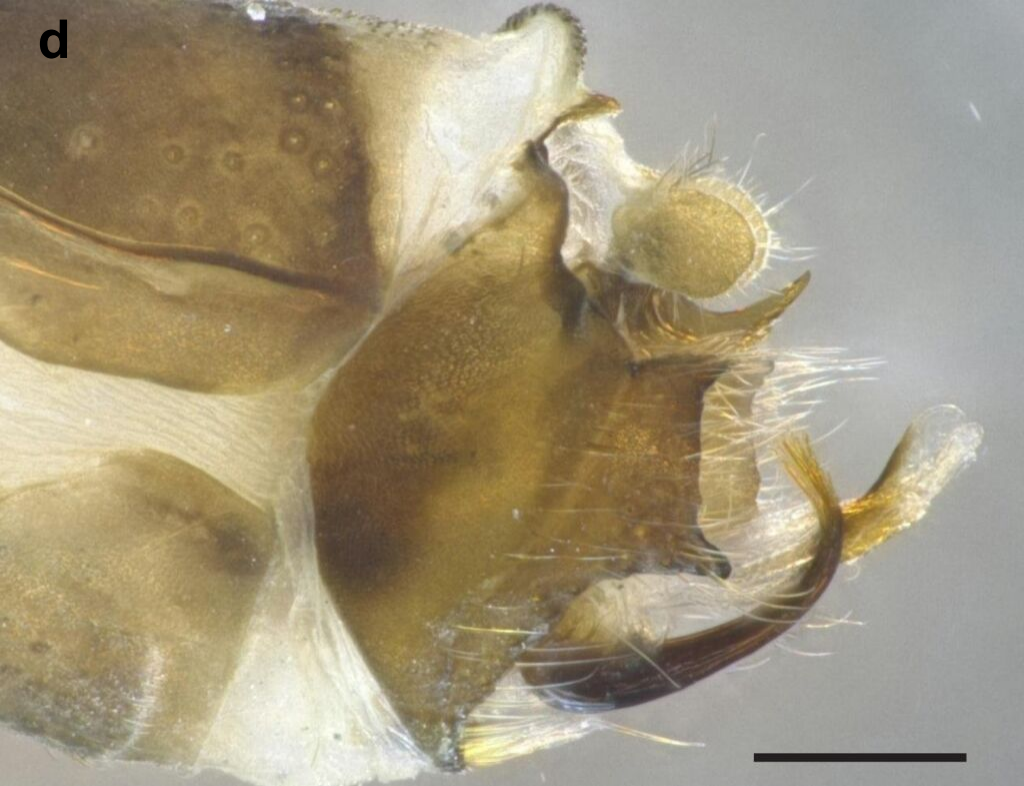
*Potamophylaxjuliani* Kumanski, 1999.

**Figure 8a.**
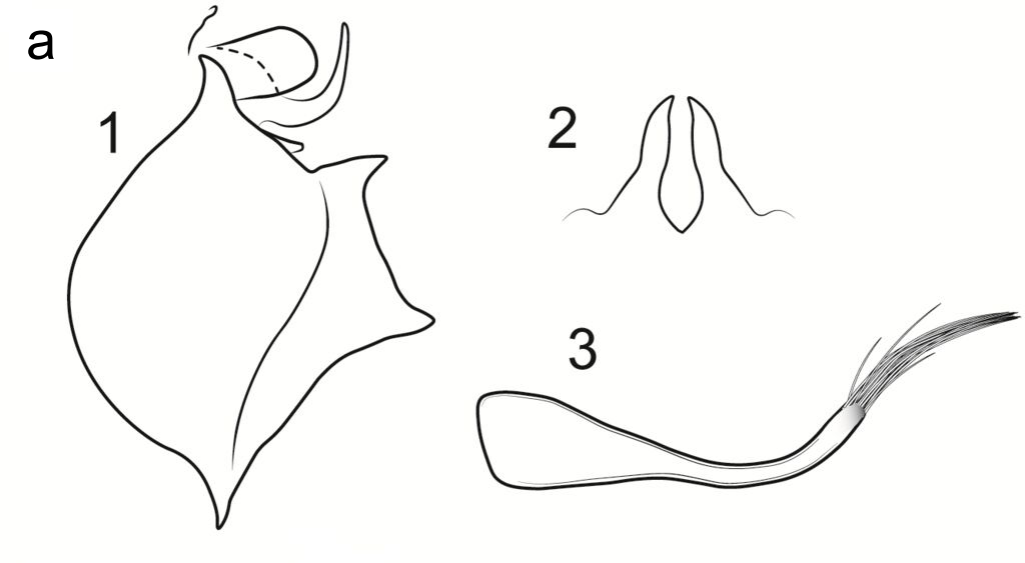
*Potamophylaxhumoinsapiens* sp. n.

**Figure 8b.**
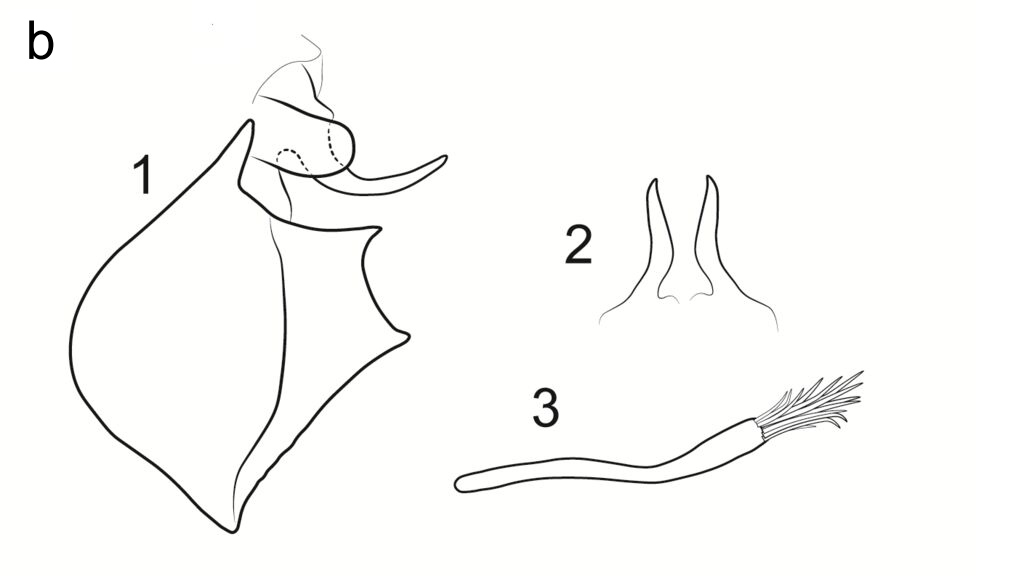
*Potamophylaxidliri* Ibrahimi, Bilalli & Kučinić, 2022.

**Figure 8c.**
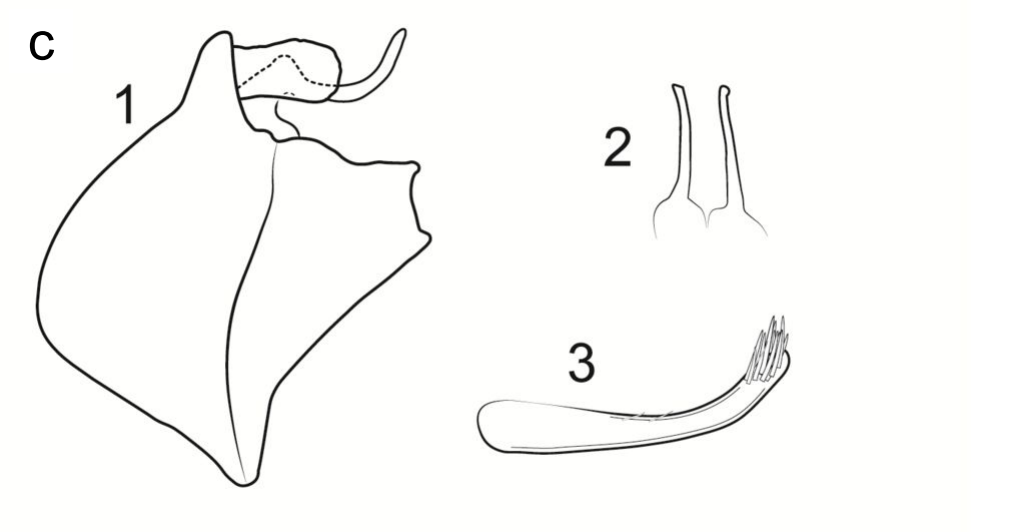
*Potamophylaxcoronavirus* Ibrahimi, Bilalli & Vitecek, 2021.

**Figure 8d.**
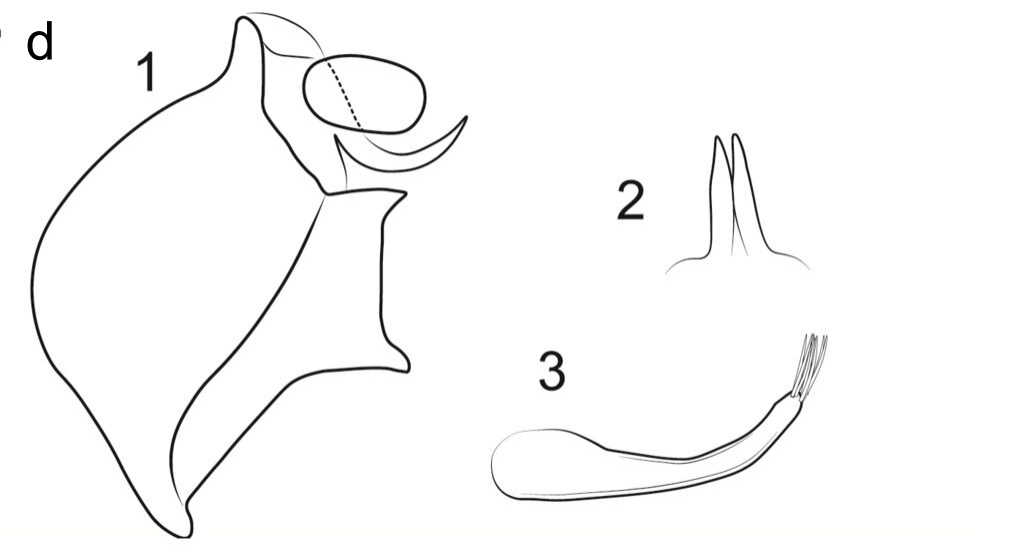
*Potamophylaxjuliani* Kumanski, 1999.

## References

[B8344298] Grapci-Kotori L., Geci D., Naumova M., Ibrahimi H., Bilalli A., Musliu M., Gashi A., Kasumaj E. (2022). Spiders from Sharr Mountain - new faunistic data (Arachnida: Araneae. Natura Croatica.

[B8344287] Halilaj H., Kupe L., Bajrami A., Icka P., Mala X., Damo R. (2021). Endemic plants in the flora of Shutman (Sharri Mountain), Kosovo - an analysis of phytogeographical elements and life forms. Natura Croatica.

[B8087725] Ibrahimi Halil, Kučinić Mladen, Gashi Agim, Grapci-Kotori Linda, Vučković Ivan, Cerjanec Darko (2012). The genus *Rhyacophila* Pictet, 1873 (Insecta: Trichoptera) in Kosovo. Aquatic Insects.

[B8087788] Ibrahimi Halil, Kučinić Mladen, Gashi Agim, Grapci-Kotori Linda (2014). Trichoptera Biodiversity of the Aegean and Adriatic Sea Basins in the Republic of Kosovo. Journal of Insect Science.

[B8087830] Ibrahimi H., Gashi A., Grapci-Kotori L., Zhushi-Etemi F., Bilalli A., Musliu M. (2015). New distribution and species records of caddisflies (Insecta: Trichoptera) from the Republic of Kosovo. Entomological News.

[B8087841] Ibrahimi Halil, Vitecek Simon, Previšić Ana, Kučinić Mladen, Waringer Johann, Graf Wolfram, Balint Miklos, Keresztes Lujza, Pauls Steffen (2016). *Drusussharrensis* sp. n. (Trichoptera, Limnephilidae), a new species from Sharr National Park in Kosovo, with molecular and ecological notes. ZooKeys.

[B8087698] Ibrahimi Halil, Vehapi Valmir (2017). Contribution to the knowledge of the caddisfly fauna (Insecta: Trichoptera) of the Sharr Mountains in Kosovo. Journal of the Kansas Entomological Society.

[B8087896] Ibrahimi Halil, Kuçi Ruzhdi, Bilalli Astrit, Musliu Milaim, Gashi Arben, Sinani Naman, Emërllahu Besnik (2019). Distribution of two rare taxa of caddisflies (Trichoptera: Rhyacophilidae, Polycentropodidae) from the Republic of Kosovo.. Biodiversity Data Journal.

[B8087908] Ibrahimi Halil, Kuçi Ruzhdi, Bilalli Astrit, Musliu Milaim, Vehapi Valmir, Gashi Agim, Grapci – Kotori Linda, Geci Donard (2019). New additions to the caddisfly fauna (Insecta: Trichoptera) of the Sharr Mountains in Kosovo. Ecologica Montenegrina.

[B8087921] Ibrahimi Halil, Bilalli Astrit, Vitecek Simon, Pauls Steffen, Erzinger Felicitas, Gashi Agim, Kotori Linda Grapci, Geci Donard, Musliu Milaim, Kasumaj Edison (2021). *Potamophylaxcoronavirus* sp. n. (Trichoptera: Limnephilidae), a new species from Bjeshkët e Nemuna National Park in the Republic of Kosovo, with molecular and ecological notes. Biodiversity Data Journal.

[B8087936] Ibrahimi Halil, Bilalli Astrit, Kučinić Mladen, Hlebec Dora, Gashi Agim, Kotori Linda Grapci, Stojanović Katarina, Živić Ivana (2022). *Potamophylaxidliri* sp. nov. (Trichoptera: Limnephilidae), a new species from the Jastrebac Mountains in Serbia, with molecular and ecological notes. Zootaxa.

[B8087949] Karaouzas Ioannis, Ibrahimi Halil, Waringer Johann (2018). The larva of *Rhyacophilapalmeni* McLachlan 1879 (Trichoptera: Rhyacophilidae) from Greece and Kosovo with notes on ecology and zoogeography including a key to the known Greek *Rhyacophila* larvae. Zootaxa.

[B8344381] Komnenov M., Komnenov (2017). New data on spider fauna (Araneae) of Shar Mountain, North-Western Macedonia. Proceedings of the 5th Congress of the Ecologists of Macedonia.

[B8087958] Kumanski K., Malicky H. (1999). A survey of the genus *Potamophylax* Wallengren 1891 in the Balkan Peninsula, with description of two new species (Trichoptera: Limnephilidae). Braueria.

[B8087992] Malicky H. (1986). Beschreibung von vier neuen Kocherfliegen-Arten aus der Turkei und aus Jugoslawien (Trichoptera). Opuscula Zoologica Fluminensia (Flums, Schweiz).

[B8087984] Malicky Hans (2004). Atlas of European Trichoptera / Atlas der Europäischen Köcherfliegen / Atlas des Trichoptères d’Europe.

[B8088010] Marinković-Gospodnetić M. (1975). Fauna Trichoptera SR Srbija. Zbornik Radova o Entomofauni Srbije.

[B8088019] Marinković-Gospodnetić M. (1980). Fauna Trichoptera SR Srbija. Zbornik Radova o Fauni Srbije, SANU.

[B8088028] Ministry of Environment and Spatial Planning Republic of Kosovo (2015). Sharri National Park, Management Plan 10-Year Management Strategy 2015-2024. 94 pp.

[B8088036] Ministry of Environment and Spatial Planning Republic of North Macedonia (2021). The draft law for the declaration of a part of Mountain Sharr as a national park. 29 pp.

[B8088044] Morse J. C. Trichoptera World Checklist. http://entweb.clemson.edu/database/trichopt/index.htm.

[B8088075] Nielsen A. (1957). A comparative study of the genital segments and their appendages in male Trichoptera.

[B8344428] Oláh J., Kovács T. (2012). New species and records of autumnal Trichoptera from Albania. Folia Historico Naturalia Musei Matraensis.

[B8088735] Oláh J., Kovács T. (2013). New species and records of Balkan Trichoptera II. Folia Historico Naturalia Musei Matraensis.

[B8088877] Pongrácz S. (1923). Recésszárnyúak. Neuropteroiden. In: Csiki Erno Állattani Kutatásai Albániában. Explorationes zoologicae ab E. Csiki in Albania peractae. IX. A.. Magyar Tudományos Akadémia Balkán-Kutatásainak Tudományos Erdményei.

